# A remarkable new species of *Euragallia* from Peru (Insecta, Hemiptera, Cicadellidae, Agalliini), including the description of a peculiar structure of the male genitalia

**DOI:** 10.3897/zookeys.178.3038

**Published:** 2012-03-29

**Authors:** Luiz G. Rodrigues, Ana Clara Gonçalves, Gabriel Mejdalani

**Affiliations:** 1 Departamento de Entomologia, Museu Nacional, Universidade Federal do Rio de Janeiro, Quinta da Boa Vista, São Cristóvão, 20940-040, Rio de Janeiro, RJ, Brasil; 2 Pós-graduação em Zoologia, Museu Nacional, Universidade Federal do Rio de Janeiro; 3 Departamento de Zoologia, Instituto de Biologia, Universidade Federal do Rio de Janeiro, Caixa Postal 68044, 21941-971, Rio de Janeiro, RJ, Brasil

**Keywords:** Leafhopper, Megophthalminae, Membracoidea, morphology, South America, taxonomy

## Abstract

A new species of *Euragallia* Oman, 1938 from Peru (Pasco Department) is described and illustrated. *Euragallia batmani* **sp. n.** can be distinguished from the other species of the genus by the very posteriorly pronounced male pygofer, with an apical hook-like projection, and by the well-developed dorsal area of the aedeagal base, resembling the open wings of a bat. With the addition of *Euragallia batmani* sp. n., the number of *Euragallia* species is increased to 21. Only one additional species of the genus is recorded from Peru (*Euragallia prion* Kramer, 1976). A comparison between the new species and *Euragallia prion* is provided. A conspicuous structure, which connects the subgenital plates to the styles, is described in detail and named.

## Introduction

The subfamily Megophthalminae comprises approximately 53 genera and 650 species distributed in four tribes. In the Neotropical Region, about 22 genera and 280 species are known. The tribes are distributed as follows: Adelungiini has a Palearctic distribution; Megophthalmini, African and Holarctic; Evansiolini, restricted to Chile (Juan Fernandez Islands); and Agalliini (traditionally treated as a separate subfamily), cosmopolitan. The latter tribe, which is the largest within the subfamily and includes the genus herein treated, was historically studied mostly by Oman (1933, 1934, 1938a), Linnavuori (1954), and Kramer (1964, 1976). Species in this group occur primarily in dense, low-growing herbaceous or shrubby vegetation (Gonçalves and Nielson 2011).

*Euragallia* Oman, 1938 (type-species *Agallia furculata* Osborn, 1923) appears to be limited to the tropical and subtropical regions of the Western Hemisphere, being recorded from Mexico to Brazil (Kramer 1964). Oman (1938a) carried out a revision of the genus. Other meaningful contributions were made by Kramer (1964, 1976), Linnavuori (1968), Cwikla and DeLong (1985), Dutra and Coelho (1992), Nielson and Godoy (1995), and Gonçalves and Zanol (2010). As a result of these studies, *Euragallia* contains currently 20 described species (Gonçalves and Zanol 2010).

The most relevant diagnostic characters of *Euragallia* are as follows: (1) robust species, their coloration being brown or fuscous, never with bright spots; (2) crown shorter on median region than next to eyes; (3) eyes usually bulbous; (4) subgenital plates always fused to each other at least basally, frequently small, exposing apexes of styles; (5) styles never forked; (6) abdominal segment X usually well-developed; (7) seventh sternite of female reduced, sometimes exposing base of ovipositor (Linnavuori 1954, Kramer 1964, Gonçalves and Zanol 2010). In a recently published paper, Gonçalves and Zanol (2010) recognized three groups into which species of *Euragallia* can be segregated: *declivata*, *magnicauda*, and *major*.

In this paper, a new species of *Euragallia* is described and illustrated based on three males collected in Peru (Pasco Department). This is the second species of the genus recorded from Peru. The other recorded species is *Euragallia prion* Kramer, 1976. A brief discussion about similarities and differences between the new species and *Euragallia prion* is included. The new species has remarkable male genital features, including a peculiar structure connecting the subgenital plates to the styles, which is herein described.

## Material and methods

The studied specimens belong to the Museo de Historia Natural, Universidad Nacional Mayor de San Marcos (MUSM; Lima), Illinois Natural History Survey (INHS; Champaign), and Museu Nacional, Universidade Federal do Rio de Janeiro (MNRJ; Rio de Janeiro). Label data are given inside quotations with a reversed virgule (\) separating lines. Morphological terminology follows mainly Oman (1933, 1938b), except for the head (Hamilton 1981, Mejdalani 1998) and leg chaetotaxy (Davis 1975). Techniques for preparation of male genital structures follow Oman (1949). The dissected parts are stored in microvials with glycerin. The photographs of the body and of the face were prepared with the Automontage software (Synoptics Inc., Frederick, Maryland, USA) using a digital camera attached to a stereomicroscope.

## Results

### Euragallia batmani

Rodrigues, Gonçalves & Mejdalani sp. n.

urn:lsid:zoobank.org:act:6FFFBBDF-C9AD-4DBE-9378-AC942304551A

http://species-id.net/wiki/Euragallia_batmani

Figs 1 –11

#### Length.

Male holotype, 6.1 mm; male paratypes, 6.4–6.5 mm (n = 2).

#### Holotype description.

**Figures 1–6.**
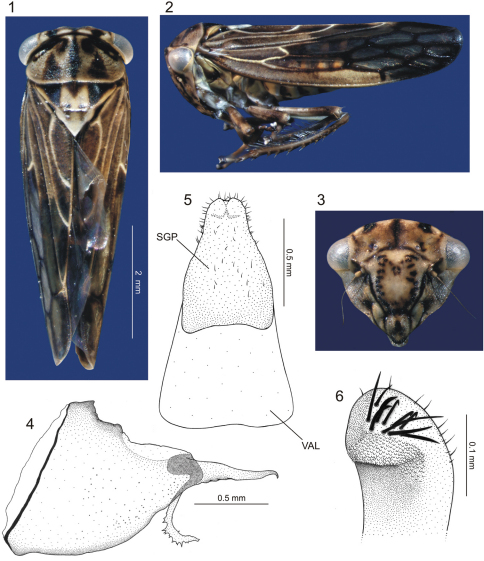
*Euragallia batmani* sp. n., body and external male genitalia. **1** body, dorsal view **2** body, lateral view **3** face, frontal view **4** pygofer, lateral view **5** valve (VAL) and (fused) subgenital plates (SGP), ventral view **6** apical portion of subgenital plate, laterodorsal view.

**Head and thorax.** Ground color of dorsum mostly yellow to brown. Crown, in dorsal view (Fig. 1), distinctly shorter medially than next to eyes; each side with fovea closer to inner eye margin than to median coronal line, each fovea with adjacent black macula; median portion of crown with black spot. Face (Fig. 3) with distinct inverted Y-shaped dark brown macula; surface depressed medially; distance between ocelli greater than distance between each ocellus and adjacent inner eye margin; ocelli slightly closer to median line of face than to adjacent inner eye margin, each ocellus with adjacent fovea and small, inconspicuous dark brown macula. Frons (Fig. 3) mostly yellow; lateral margins bordered by black line; disk with somewhat M-shaped figure formed by numerous small dark brown maculae. Genae (Fig. 3) mostly yellow, with one dark brown macula adjacent to lorum and another adjacent to antennal base; surface striated. Clypeus (Fig. 3) with transverse dark brown line at base and median dark brown macula; with small apical setae. Pronotum (Fig. 1) with pair of black-marked depressions on anterior margin; disk weakly punctured and finely transversely striated; with pair of large dark brown triangular maculae on posterior half, dark brown longitudinal line extending from anterior to posterior margin on middle portion, and dark brown macula between apex of each triangular macula and median line. Basal portion ofmesonotum (Fig. 1) with triangular dark brown macula located medially and pair of larger, lateral dark brown triangular maculae, these three maculae delimiting yellow, somewhat M-shaped area; pair of dark brown spots close to transverse sulcus. Forewings (Figs 1, 2) opaque on basal third and along costal area until base of first apical cell, remainder of surface translucent; venation pronounced; claval veins almost entirely marked with pale yellow; corium veins marked with pale yellow on basal third, remainder mostly dark brown; claval apex marked with pale yellow.

**Metathoracic leg chaetotaxy.** Row I of tibia with ten primary setae, equal in size and shape, bases slightly elevated. Row II with seven primary setae, bases elevated. Row III with eight primary setae, most proximal one almost aligned with third seta of row II. Row IV with approximately 25 primary elongate setae, increasing in size towards tibia apex; intercalary setae present; distal setae indistinct from the others of the row. Apex of tibia with distal transverse row formed by conspicuous spurs. Plantar surface of first tarsomere with small setae uniformly distributed; apex bearing one conspicuous platella.

**Figures 7–11.**
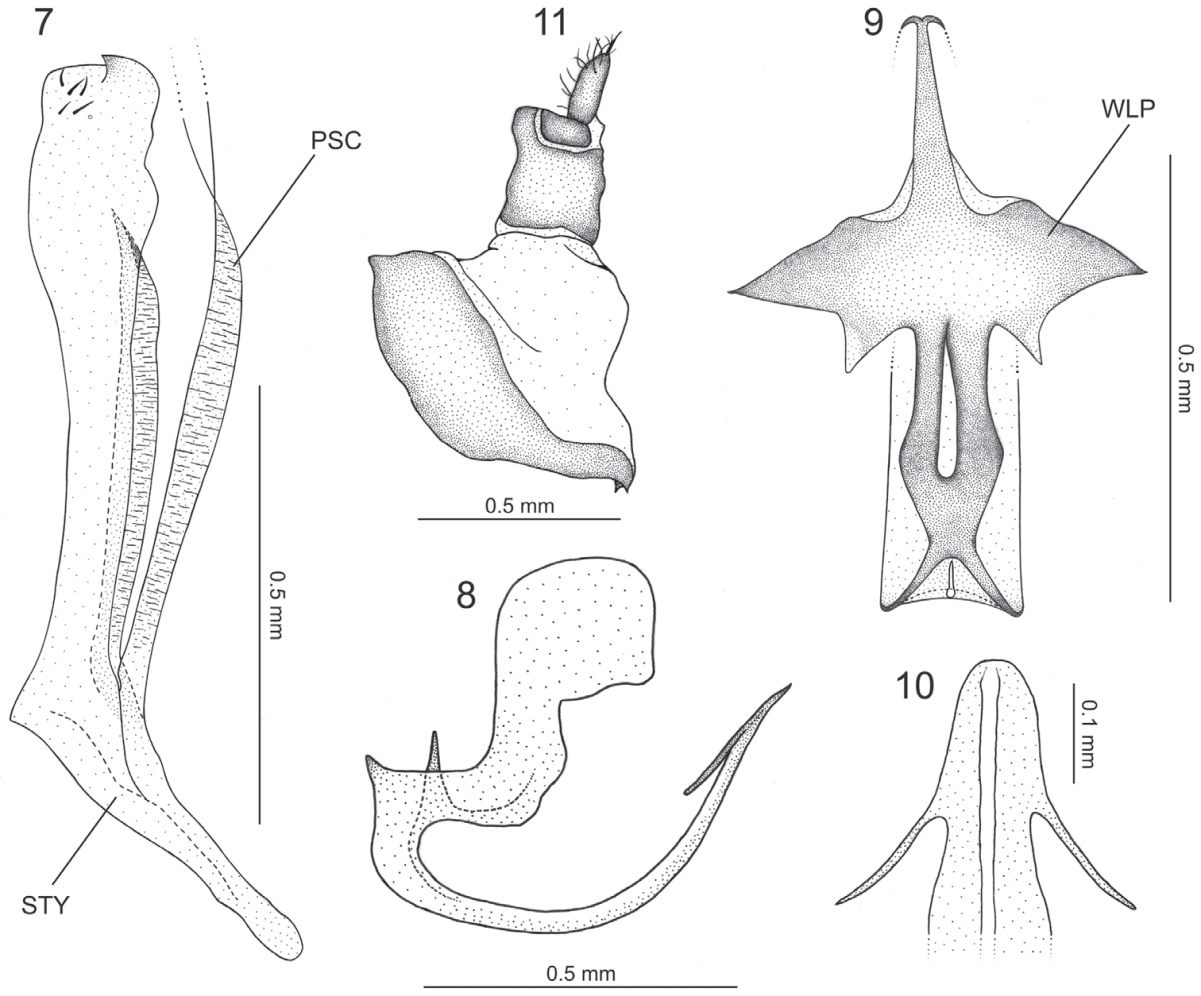
*Euragallia batmani* sp. n., internal male genitalia and anal tube. **7** style (STY), dorsal view (PSC = plate-style connective) **8** aedeagus, lateral view **9** dorsal region of basal portion of aedeagus, dorsal view (WLP = wing-like projection) **10** apical portion of aedeagus, caudal view **11** anal tube, lateral view.

**Male genitalia.** Pygofer (Fig. 4), in lateral view, pronounced posteriorly, forming very long projection with about half of pygofer length; apex of projection hook-like; inner dorsal margin with elongate process extending ventrally and then gradually curved posteriorly, armed apically with irregular dentiform projections. Valve (Fig. 5) trapezoidal, fused to subgenital plates. The latter (Fig. 5), in ventral view, completely fused to each other, subtriangular, with slight apical emargination; surface with small scattered setae; apical portion, in lateral view, curved dorsally, with some well-developed setae and scale-like sculpture (Fig. 6). Connective membranous. Styles (Fig. 7), in dorsal view, expanded on apical third, club-shaped, bearing small apical claw-like projection directed inward; basal portion of style with elongate sclerotized projection, which is fused apically to the dorsal apical portion of subgenital plate and lays on a groove on outer style margin (Fig. 7 – PSC [*plate-style connective*]). Aedeagus (Fig. 8) symmetrical; shaft, in lateral view, strongly flattened dorsoventrally, base directed ventrally, then gradually curved dorsally; apical portion directed posterodorsally, bearing pair of subapical, slender lateral spiniform processes (Fig. 10) extending anteriorly for short distance; base of aedeagus, in dorsal view, with dorsal region well-developed, bearing lateral sclerotized projections resembling the open wings of a bat (Fig. 9 – WLP); base of aedeagus (Fig. 8), in lateral view, with spiniform projection directed dorsally. Segment X (Fig. 11), in lateral view, strongly sclerotized, bearing pair of small apical processes on each side.

**Female unknown.**

#### Intraspecific variation.

The number of primary setae on the rows of the metathoracic tibia may vary slightly; for instance, the number of setae on row III may be eight (holotype) or nine (one paratype). Additional cross veins may be present on the forewings; for instance, the median anteapical cell may be divided by a cross vein. The basal portion of the forewings may be more translucent than in the holotype.

#### Etymology. 

The specific epithet, *batmani*, is a reference to the dorsal region of the aedeagal base in dorsal view (Fig. 9), which closely resembles the open wings of a bat, like those of the Batman symbol.

#### Type material.

Peru, Pasco Department. Holotype: male, “Peru: Pasco, Yanachaga-Chemil- \ lén N.P., Huampal Stn. 10°11'9"S, 75°34'27"W, 1050m, 6–9 X 2002 \ D. Takiya, C. Peña, R. Rakitov \ Malaise trap acr. R. Huancabamba” (MUSM). Paratypes: two males with same data as the holotype (INHS, MNRJ).

## Discussion

Among the *Euragallia* species, *Euragallia prion* seems to be the most similar to *Euragallia batmani* sp. n. In Gonçalves and Zanol (2010), the new species keys to the couplet of *Euragallia prion*, and according to their proposal of species groups, it belongs in the *major* group, as well as *Euragallia prion*. The male pygofer of both species has the posterodorsal process armed with similar dentiform projections. The two species also have a pair of lateral subapical processes on the aedeagal shaft. Nevertheless, the new species can be easily distinguished from *Euragallia prion* by the shape of the posterior margin of the pygofer, which is very pronounced posteriorly in the former (Fig. 4). Such pygofer projection is absent in *Euragallia prion*. The bat-like shape of the dorsal region of the aedeagal base (Fig. 9 – WLP) is another character that clearly distinguishes *Euragallia batmani* from *Euragallia prion* and the remaining species of the genus.

The basal portion of the styles of the new species bears a conspicuous, sclerotized elongate projection, which lays on a groove on the outer style margin. This projection, which is fused apically to the dorsal apical portion of the subgenital plate, is herein named the *plate-style connective* (Fig. 7 – PSC). This structure was previously recorded in only three species of the genus, *Euragallia goemansi* Gonçalves & Zanol, 2010, *Euragallia adelinae* Gonçalves & Zanol, 2010, and *Euragallia nervata* (Oman, 1934), being described and illustrated in the first two by Gonçalves and Zanol (2010) and illustrated only in the last by Linnavuori and DeLong (1979). Despite the fact that the PSC is recorded in the literature for only three species, the present authors have noticed that the majority of the species within the genus possess this structure. We believe that future studies will point out the relevance of the PSC for the taxonomy of *Euragallia*. The function of the PSC is not known. It is also not known whether the development of the PSC is morphofunctionally associated with the reduced (membranous) connective of the new species or whether the two conditions arose independently.

## Supplementary Material

XML Treatment for Euragallia batmani
